# Clinical evaluation of dengue and identification of risk factors for severe disease: protocol for a multicentre study in 8 countries

**DOI:** 10.1186/s12879-016-1440-3

**Published:** 2016-03-11

**Authors:** Thomas Jaenisch, Dong Thi Hoai Tam, Nguyen Tan Thanh Kieu, Tran Van Ngoc, Nguyen Tran Nam, Nguyen Van Kinh, Sophie Yacoub, Ngoun Chanpheaktra, Varun Kumar, Lucy Lum Chai See, Jameela Sathar, Ernesto Pleités Sandoval, Gabriela Maria Marón Alfaro, Ida Safitri Laksono, Yodi Mahendradhata, Malabika Sarker, Firoz Ahmed, Andrea Caprara, Bruno Souza Benevides, Ernesto T. A. Marques, Tereza Magalhaes, Patricia Brasil, Marco Netto, Adriana Tami, Sarah E. Bethencourt, Maria Guzman, Cameron Simmons, Nguyen Thanh Ha Quyen, Laura Merson, Nguyen Thi Phuong Dung, Dorothea Beck, Marius Wirths, Marcel Wolbers, Phung Khanh Lam, Kerstin Rosenberger, Bridget Wills

**Affiliations:** Section Clinical Tropical Medicine, Heidelberg University Hospital, Heidelberg, Germany; Oxford University Clinical Research Unit, 764 Vo Van Kiet Street, District 5, Ho Chi Minh City, Vietnam; University of Medicine and Pharmacy of Ho Chi Minh City, Ho Chi Minh City, Vietnam; Hospital for Tropical Diseases, Ho Chi Minh City, Vietnam; Children’s Hospital Number 2, Ho Chi Minh City, Vietnam; National Hospital for Tropical Diseases, Hanoi, Vietnam; Department of Medicine, Imperial College, London, UK; Angkor Hospital for Children, Siem Reap, Cambodia; University of Malaya Medical Centre, Kuala Lumpur, Malaysia; Ampang Hospital, Kuala Lumpur, Malaysia; Hospital Nacional de Niños Benjamin Bloom, San Salvador, El Salvador; Gadjah Mada University, Yogyakarta, Indonesia; James P Grant School of Public Health, BRAC University, Dhaka, Bangladesh; International Center for Diarrhoeal Diseases Research, Dhaka, Bangladesh; Universidade Estadual Do Ceará, Fortaleza, Brazil; Centro de Pesquisas Aggeu Magalhaes, Fundacao Oswaldo Cruz, Recife, Pernambuco Brazil; Instituto Nacional de Infectologia Evandro Chagas, Fundacao Oswaldo Cruz, Rio de Janeiro, Brazil; Secretaria Municipal de Saúde de Resende, Rio de Janeiro, Brazil; Department of Medical Microbiology, University of Groningen, University Medical Center Groningen, Groningen, The Netherlands; Facultad de Ciencias de la Salud, Universidad de Carabobo, Valencia, Venezuela; Institute Pedro Kouri, Havana, Cuba; Department of Microbiology and Immunology, The Peter Doherty Institute, University of Melbourne, Melbourne, Australia; Centre for Tropical Medicine and Global Health, Nuffield Department of Clinical Medicine, Oxford University, Oxford, UK; Present address: Rwanda Military Hospital and the University of Rwanda in Kigali, Kigali, Rwanda; Present address: St. Jude Children’s Research Hospital, Memphis, TN USA

**Keywords:** Dengue, Asia, Latin America, Diagnosis, Risk prediction, Pathogenesis

## Abstract

**Background:**

The burden of dengue continues to increase globally, with an estimated 100 million clinically apparent infections occurring each year. Although most dengue infections are asymptomatic, patients can present with a wide spectrum of clinical symptoms ranging from mild febrile illness through to severe manifestations of bleeding, organ impairment, and hypovolaemic shock due to a systemic vascular leak syndrome. Clinical diagnosis of dengue and identification of which patients are likely to develop severe disease remain challenging. This study aims to improve diagnosis and clinical management through approaches designed a) to differentiate between dengue and other common febrile illness within 72 h of fever onset, and b) among patients with dengue to identify markers that are predictive of the likelihood of evolving to a more severe disease course.

**Method/Design:**

This is a prospective multi-centre observational study aiming to enrol 7–8000 participants aged ≥ 5 years presenting with a febrile illness consistent with dengue to outpatient health facilities in 8 countries across Asia and Latin America. Patients presenting within 72 h of fever onset who do not exhibit signs of severe disease are eligible for the study. A broad range of clinical and laboratory parameters are assessed daily for up to 6 days during the acute illness, and also at a follow up visit 1 week later.

**Discussion:**

Data from this large cohort of patients, enrolled early with undifferentiated fever, will be used to develop a practical diagnostic algorithm and a robust clinical case definition for dengue. Additionally, among patients with confirmed dengue we aim to identify simple clinical and laboratory parameters associated with progression to a more severe disease course. We will also investigate early virological and serological correlates of severe disease, and examine genetic associations in this large heterogeneous cohort. In addition the results will be used to assess the new World Health Organization classification scheme for dengue in practice, and to update the guidelines for “Integrated Management of Childhood Illness” used in dengue-endemic countries.

**Trial registration:**

NCT01550016. Registration Date: March 7, 2012

## Background

Dengue is an arboviral disease caused by infection with any one of four related dengue virus (DENV) serotypes. It is currently the most important mosquito-borne viral pathogen affecting humans, and is emerging as a major threat to global health. Best estimates indicate that some 3 billion people live in parts of the world where they are at risk of infection and that around 96 million symptomatic episodes and approximately 20,000 deaths occur each year [1]. As yet, neither vaccines nor specific therapies are available although both areas are currently the focus of intense research efforts.

Among symptomatic dengue cases a wide variety of clinical manifestations are seen, ranging from mild febrile illness to severe and potentially fatal disease. Only a small proportion of patients progress to more severe disease, typically manifesting with a transient systemic vascular leak syndrome around the time of defervescence; plasma leakage occurs, usually accompanied by altered haemostasis and thrombocytopenia [2]. Leakage may be profound, particularly in children, sometimes resulting in life-threatening dengue shock syndrome (DSS). Other severe complications, such as severe liver, cardiac or neurological involvement, may also occur but are less frequent. With expert supportive care mortality rates have been reduced to very low levels, in many centres of excellence down to less than 1 % for those with severe disease [3]. Careful observation and judicious use of intravenous fluid therapy are crucial, with urgent shock resuscitation required in only a small proportion of cases. However, a major issue for clinicians treating such patients remains the fact that clinical diagnosis of dengue is difficult in the early febrile phase of the illness without reliance on expensive diagnostics. Secondly prediction of risk for the development of complications such as shock due to systemic vascular leak syndrome is currently poor. As a result very large numbers of patients with possible dengue, potentially at risk for severe disease, are admitted to higher level healthcare facilities in endemic areas primarily for observation, overburdening the system such that the often limited local resources are not used to maximal advantage for those patients who do need expert care.

### Clinical features and warning signs

Two major clinical syndromes, dengue fever (DF) and dengue haemorrhagic fever (DHF), were first described 50 years ago in Thailand, with case definitions and management guidelines published by the World Health Organization (WHO) in the 1970s [4]. However, practical limitations with this classification system have become increasingly apparent, and a growing body of evidence indicates that the two syndromes are part of a continuous spectrum of disease rather than being discrete entities [5–8]. As a result a revised classification system has been developed, based in part on prospectively collected data from over 2000 patients recruited across seven endemic countries (DENCO study), which was adopted in the revised WHO dengue guidelines published in 2009 [9, 10]. The new scheme classifies the disease more simply into dengue and severe dengue, with the hope that this will prove more effective for triage and clinical management, and will also improve the quality of surveillance and epidemiological data collected globally.

Dengue and other febrile illnesses (OFI) share many clinical features (e.g. headache, myalgia, rash), and most countries where dengue is common also have epidemics of illnesses such as measles, typhoid, leptospirosis and influenza that are easily confused with dengue in the early phase. Although a case definition for dengue fever has been included in the WHO guidelines for many years [11, 12], in view of the variability in clinical presentation laboratory confirmation is considered desirable. However for many endemic countries this is not a feasible option, and simple and inexpensive strategies that rely on clinical and/or readily available laboratory parameters to provide a reliable early diagnosis are needed. Clinical warning signs suggestive of likely progression to severe dengue are also included in the new guidelines, but the evidence from the DENCO study in support of specific warning signs was limited by the small numbers of patients who progressed to the severe category while under observation. Thus, although warning signs are considered a key component for early recognition of potentially severe disease, the current evidence for any clinical/laboratory markers is weak. Further research is need to determine whether clinical/laboratory warning signs with high predictive power can indeed be identified, but to do this a very large cohort of patients needs to be assessed from the early febrile phase, in order to capture sufficient patients who progress to more severe disease.

### Pathogenesis

Despite intensive efforts dengue disease pathogenesis remains incompletely understood, particularly in relation to the mechanisms responsible for the systemic vascular leak syndrome [13]. It is clear that although all four viral serotypes can cause fatal disease, second or subsequent infections are much more likely to be associated with severe clinical manifestations than primary infections [14, 15]. There is also evidence to suggest that severe disease is associated with higher plasma viremia, and to implicate immune response mechanisms to the virus as playing a significant role in pathogenesis [16, 17]. However, data on plasma viremia in primary versus secondary infections and/or in relation to disease severity are inconsistent, and detailed examination of viremia kinetics has been limited to date, with the focus primarily on hospitalised patients [18–20].

Dengue non-structural protein (NS1) is a glycoprotein expressed by dengue-infected cells [13, 21, 22]. The availability of commercial antigen-capture assays that detect NS1 in plasma or serum presents an opportunity for it to be used for early and rapid dengue diagnosis. It has also been proposed as a prognostic marker for severe disease [23]. Numerous studies have confirmed NS1 to be highly specific for dengue, but sensitivity has been variable according to the serotype involved, the geographical location, the immune status of the patient, and the day of illness on testing [24, 25]. Since it is crucial to understand the limitations of such tests before they can be recommended for routine use in the community, large, well-powered studies that can determine the true sensitivity and specificity of NS1 testing in real world settings are essential.

Dengue virus reactive IgG antibodies are also considered to play a critical role in determining risk for severe dengue via processes such as virus neutralisation and/or infection enhancement [26]. However, despite their importance the features of polyclonal sera that are associated with development of severe dengue have not been characterised as yet. Finally, although genetic predisposition to dengue has long been suggested, almost every study on this topic has been underpowered, did not consider population stratification, and did not go on to replicate the findings in independent patient cohorts. In the first genome-wide case–control genetic association study performed in dengue, several SNPs associated with DSS that are significant at the genome-wide level were identified, the most striking associations being SNPs in the MICB and PLCE1 genes [27]. Genetic variants of MICB and PLCE1 have also been found to be associated with less severe forms of dengue, and also dengue in infants [28]. Validation that these SNPs are associated with a) clinically apparent dengue (as distinct from DSS) in patients from other Asian and also Latin American countries and b) the magnitude of viremia or NS1 concentration in the early febrile period are required.

In summary, the pathological mechanisms associated with severe dengue remain obscure, and the crucial question of exactly how the virus causes the serious complications observed in patients remains unanswered.

### Primary objectives

The study aims to improve diagnosis and clinical management of dengue through approaches designed a) to differentiate between dengue and other common febrile illness within 72 h of fever onset, and b) among patients with dengue to identify markers that are predictive of the likelihood of evolving to a more severe disease course.

- AIM 1: To identify clinical and/or simple laboratory parameters which differentiate between dengue and non-dengue illness within the first 72 h of fever, with the overall aim of developing a robust case definition for dengue.
- AIM 2: To identify clinical and/or simple laboratory parameters among dengue infected patients that predict likely progression to a more severe disease course.
- AIM 3: To identify virological correlates of severe dengue – in particular to assess plasma viremia and NS1 antigenemia within the first 72 h of fever in patients with confirmed dengue.
- AIM 4: To identify early serological correlates of severe dengue, in order to characterize differences between severe and uncomplicated disease.

### Secondary Objectives

- AIM 1: To evaluate practical application of the original (WHO 1997) and the new dengue classification schemes (WHO 2009) across a series of clinical sites.
- AIM 2: To use the data to update the guidelines for “Integrated Management of Childhood Illness” used in dengue-endemic countries.

## Methods/Design

This is a prospective multi-centre observational study aiming to enrol 7 to 8,000 patients presenting with a febrile illness consistent with a diagnosis of dengue to outpatient health facilities in urban centres in 8 countries across Asia and Latin America. The study is coordinated centrally by the Section Clinical Tropical Medicine in Heidelberg, Germany, and the Oxford University Clinical Research Unit (OUCRU) in Ho Chi Minh City (HCMC), Vietnam. The countries participating in the study, and the centres coordinating the work locally in each country, are indicated in Table 1. The study is being performed in accordance with ICH-GCP guidelines, with regular oversight by a team of independent monitors.

**Table 1:** Countries/centres enrolling participants into the study

Coordinating Centre	City, Country	Ethics Committees Involved
Heidelberg University Hospital	San Salvador, El Salvador	• Comité de Ética en Investigación Clínica (CEIC), Hospital Nacional de Niños Benjamín Bloom, San Salvador, El Salvador
Ethics Committee, Faculty of Medicine, Heidelberg University	Fortaleza, Brazil	• Comitê de Ética em Pesquisa da Universidade Estadual do Ceará (CEP-UECE), Fortaleza, Brazil • Comissão Nacional de Ética em Pesquisa (CONEP), Brasília, Brazil
	Recife, Brazil	• Comitê de Ética em Pesquisa do Centro de Pesquisas Aggeu Magalhães (CEP-CPqAM) • Comitê de Ética em Pesquisa em Seres Humanos do Instituto de Medicina Integral Prof. Fernando Figueira (CEP-IMIP), Recife, Brazil • Comissão Nacional de Ética em Pesquisa (CONEP), Brasília, Brazil
	Rio de Janeiro, Brazil	• Instituto de Pesquisa Clínica Evandro Chagas (IPEC), Fundação Oswaldo Cruz (Fiocruz), Rio de Janeiro, Brazil • Comissão Nacional de Ética em Pesquisa (CONEP), Brasília, Brazil
	Maracay, Venezuela	• Comité de Bioética del Instituto de Investigaciones Biomédicas de la Universidad de Carabobo, Maracay, Venezuela
	Dhaka, Bangladesh	• Ethical Review Committee, International Centre for Diarrhoeal Disease Research, Dhaka, Bangladesh
Oxford University Clinical Research Unit – Viet Nam	Yogyakarta, Indonesia	• Medical and Health Research Ethics Committee, Faculty of Medicine, Gadjah Mada University, Yoygakarta, Indonesia
Oxford Tropical Research Ethics Committee (OxTREC)	Siem Reap, Cambodia	• Institutional Review Board, Angkor Hospital for Children, Siem Reap, Cambodia
	Kuala Lumpur, Malaysia	• Medical Ethics Committee, University of Malaysia Medical Center, Kuala Lumpur, Malaysia • Medical Research and Ethics Committee, Ministry of Health, Kuala Lumpur, Malaysia
	Ho Chi Minh City, Viet Nam	• Ethics Committee, Children’s Hospital Number 2, Ho Chi Minh City, Viet Nam • Ethics Committee, Hospital for Tropical Diseases, Ho Chi Minh City, Viet Nam
	Hanoi, Viet Nam	• Ethics Committee, National Hospital for Tropical Diseases, Hanoi, Viet Nam

### Study population and patient enrolment

Both adults and children (≥5 years) are eligible for enrolment, and it is anticipated that the relative proportions will reflect the expected local dengue epidemiology in each country. Following appropriate informed consent, subjects presenting to outpatient departments (OPD) at the designated sites with fever for ≤ 72 h without localizing features, i.e. consistent with a possible diagnosis of dengue, are being enrolled.

### Inclusion criteria

Any patient presenting to the OPD at the participating centres is eligible for enrolment if they meet the following criteria:

- Age ≥ 5 years
- Fever or history of fever for ≤ 72 h
- Clinical symptoms consistent with possible dengue – i.e. suspected dengue and/or undifferentiated fever in a patient from a dengue endemic area
- Considered by the treating physician to be suitable for outpatient care at the time of study enrolment – i.e. no signs of severe disease
- Written informed consent

### Exclusion criteria

- Localizing features suggesting an alternative diagnosis, eg pneumonia, otitis etc.
- The physician judges that the patient is unlikely to attend daily follow up - e.g. due to travelling distance from the clinic

At each OPD a triage system has been established to direct potentially eligible subjects to a selected outpatient room/area for consideration for enrolment, where study staff are available to assist the regular clinic staff in patient enrolment and collection of data. The study staff discuss the study with all potential adult participants or, in the case of children 5–17 years of age accompanied by a parent/guardian, with the accompanying parent/guardian. Study staff describe the purpose of the study, the study procedures, possible risks/benefits, the rights and responsibilities of participants, and alternatives to enrolment, and provide a written Patient Information Sheet. If the patient or parent/guardian agrees to participate, they are asked to sign an Informed Consent Form, a copy of which is then given to them to keep. If the patient or parent/guardian is indecisive about enrolment, they are given as much time as necessary to consider the study up until 72 h of illness has passed at which point the patient/child is no longer eligible for the study. Children aged 12–17 are also asked to give assent to the study, after discussion with study staff and having their questions answered. A separate Assent Form is provided, for the child to sign with a copy for them to keep. In addition to the procedures above, illiterate signatories have the Informed Consent/Assent Form read to them in the presence of a witness who signs to confirm this. All Patient Information Sheets and Consent/Assent forms are written in the local language and use terms that are easily understandable.

### Data collection instrument

A structured clinical questionnaire is completed upon enrolment and then once daily for up to six days for all patients in the study. This case report form (CRF) includes detailed clinical signs and symptoms, as well as all standard laboratory results. Data collection focuses in particular on recording the timing of onset of new signs or symptoms, and on assessing the severity of symptoms in a systematic way (mild/moderate/severe). The final format was agreed by the Principal Investigators at the participating clinical centres prior to translation into relevant languages for local use. The CRF is supported by a series of standard operating procedures (SOPs) describing in detail all study procedures, severity definitions and methods for documentation, and all SOPs are also presented in the appropriate local language.

### Clinical evaluation

Following enrolment clinical history and examination findings are recorded in the CRF, and a 3–5 ml (age-dependent) research blood sample is obtained, together with appropriate samples to measure a range of haematological and biochemical parameters following local laboratory requirements (Table 2). Patients are then reviewed daily in the OPD until fully recovered and afebrile for 24 h, or for up to 6 days from enrolment. A rapid access card is provided to each study participant to facilitate these daily visits. Standardized clinical information is recorded and a full blood count is performed at each visit, plus any other tests deemed necessary by the clinic physician. On the last acute illness visit (within approximately 24 h of defervescence) a second sample for a biochemical profile is obtained together with a sample for serology. All patients are then asked to attend a final follow-up visit around day 10–14 of illness, at least one week from the last visit during the acute illness. In addition to a clinical assessment at this time a last blood sample is taken for convalescent serology and a full blood count.

**Table 2:** Blood sample collection - schedule and volumes

	Test	Enrolment (≤72 h fever)	Daily for up to 6 days	Final acute visit	Follow-up, Day 10-14
Research	Dengue NS1 Dengue IgM/G DENV PCR Serological & virological and/or genetic studies	3 ml EDTA for children 5 ml EDTA for adults		2 ml EDTA	2 ml EDTA
Haematology	Full blood count	1 ml EDTA	1 ml EDTA	1 ml EDTA	1 ml EDTA
Biochemistry	AST/ALT Albumin Creatine kinase Creatinine	2 ml Lithium Heparin^a^		2 ml Lithium Heparin^a^	

^a^or serum/an alternative additive according to local laboratory requirements

All management decisions throughout the acute illness are at the discretion of the clinic physicians. Any patient requiring hospital admission continues to be followed daily using a similar but more detailed CRF, with the indication(s) for admission clearly documented, and all management interventions recorded together with the physician’s rationale for these interventions.

### Laboratory evaluation

Haematology and biochemistry profiles are performed on standard laboratory instruments at the various participating sites, with evidence for quality assurance and quality control checked by study monitors at regular intervals.

NS1 antigen detection (Platelia NS1, Biorad) and diagnostic serological assays (IgM and IgG Capture ELISA Kits, Panbio, Australia) are being performed in batches at a designated laboratory in each country, or at the OUCRU laboratory in HCMC if in-country diagnostics are not feasible. Proficiency panels were used to assess the capacity of each site to perform the assays in an appropriate manner before the study commenced. NS1 detection is attempted on all enrolment plasma samples, while diagnostic serology is performed on acute and convalescent plasma samples (follow-up visit specimen if available, or final acute visit specimen if not).

Plasma viremia levels are measured in designated centres on enrolment plasma samples by qRT-PCR using a validated assay. Samples from all sites in Vietnam, Cambodia, Malaysia, Bangladesh, and El Salvador are being assayed at the OUCRU laboratory in HCMC. The research laboratory at the Gadjah Mada University is responsible for the Indonesian diagnostics, while specimens from the remaining sites in Latin America will be assayed at the FIOCRUZ Laboratories in Recife and Rio de Janeiro, or at the Institute Pedro Kouri in Havana, Cuba. Harmonization of assay protocols, reagents and equipment has been performed to ensure equivalence between the laboratories. A single diagnostic database is maintained in HCMC, where data from all sites are collated and checked for errors/inconsistencies following a formal data validation SOP.

For the detailed serological research studies, a range of high-throughput functional and serological assays will be considered for the measurement of DENV-specific antibodies. The intention of the serological studies will be to understand whether the quality and quantity of the anti-DENV antibody response differs between patients with mild versus severe clinical or laboratory outcomes.

### Case definitions

**Fig. 1:**
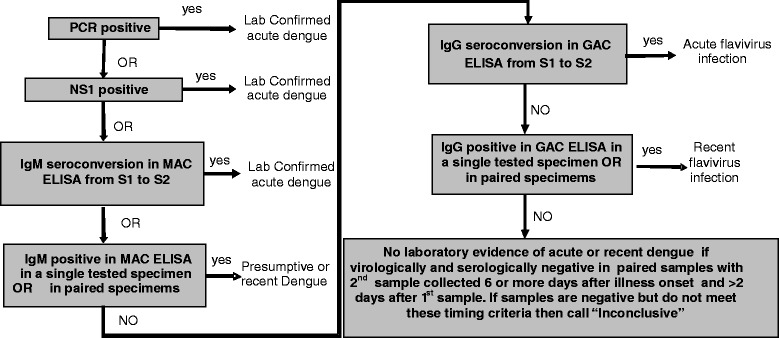
Diagnostic Algorithm. PCR and NS1 results are obtained from analyzing the enrolment sample while IgM and IgG results are obtained from analyzing an acute phase sample (S1) and a follow-up/early convalescent phase sample (S2). In the presence of a clinical syndrome that might be dengue, a positive result on any of the first 3 tests is sufficient for a diagnosis of “laboratory confirmed acute dengue”

Laboratory diagnosis of a dengue case will be in accordance with current WHO criteria [9]. The diagnostic algorithm is shown in Fig. 1. Briefly, any case with virological evidence of dengue as shown by a positive RT-PCR assay or NS1 ELISA test, or who has IgM seroconversion between paired specimens, is defined as having laboratory-confirmed dengue. A diagnosis of OFI is assigned to participants with no laboratory evidence of acute or recent dengue. Among participants with confirmed dengue a probable primary infection is defined by negative IgG Capture results on both the enrolment and follow-up samples, while a probable secondary infection is defined by a positive IgG Capture result in either or both the enrolment and follow-up samples.

### Data management

A customized data entry software tool was developed consisting of a Microsoft Access database with a graphical user interface which follows the layout of the paper CRFs. An import function for laboratory results is built into the data entry tool in order to facilitate automatic uploads at the sites where this is technically feasible. The laboratory section of the paper CRF as well as the data entry software tool was adapted to site-specific units in order to avoid conversion errors during data entry. The data entry software is installed on designated computers with restricted access. Entered data is stored locally at the site and can be edited until uploaded to a secure server at the coordinating centre in Heidelberg. Uploads are automatically integrated into the central database. The data of the central server is mirrored to a secure backup server.

Double data entry is carried out for 100 % of the first 100 patients enrolled at each site, then gradually reducing to a minimum of 5 % double data entry depending on the proportion of discrepancies between 1^st^ and 2^nd^ entries. Plausibility checks as well as range checks are built into the data entry tool, so that the data quality is checked in real time during data entry. Once the data is uploaded to the central server, the quality is checked again for plausibility and missing values by pre-programmed algorithms. The resulting query reports are relayed back to the local sites for correction before the data is again uploaded and processed for analysis.

Data and study materials will be stored for a minimum of 5 years at each site after the completion of the project. Individual SOPs will be written for continued storage or destruction according to local requirements and the availability of facilities for long-term storage. After analysis, the coordinating centre in Heidelberg will archive the database for long-term storage.

### Clinical monitoring

Independent monitoring has been integrated into the programme of work from the outset to ensure full compliance with Good Clinical Practice standards and all regulatory requirements. Monitoring teams from the OUCRU Clinical Trials Unit travel intermittently to all study sites in Southeast Asia, while a team from Heidelberg is responsible for the Latin American sites and Bangladesh. Following a site initiation visit, the first monitoring visit takes place after 50–100 participants have enrolled, followed by regular visits (at least annually) depending on recruitment rates. The monitors review study eligibility and informed consent for 100 % of the enrolled subjects. Data accuracy for all clinical information and all routine laboratory results (haematology and biochemistry) is also reviewed for 100 % of all participant CRFs during the first 6 months of enrolment at each site. Thereafter this percentage is maintained or decreased based on the error rate found during the most recent monitoring visit. At the end of each visit, the monitoring team meets with the site principal investigator and relevant study staff to review and present any findings, and on subsequent monitoring visits the teams review changes made in response to previous findings/recommendations.

### Statistical methods and analysis of results

The statistical methodology to be used for the primary objectives is summarised briefly in the following section. Detailed analysis plans will be formulated and documented prior to commencement of particular sections of the analysis relating to specific aims.

#### Sample size

We anticipate that dengue will be confirmed in ~ 50 % of the patients enrolled in Asia and ~ 20–30 % of the patients enrolled in Latin America. We also expect to make a definite negative diagnosis – i.e. that the patient did not have dengue – in at least 80 % of the remaining patients. Among hospitalised patients enrolled in previous descriptive studies in Vietnam around 5 % of cases progressed to severe disease; there is currently no data available for patients managed in the community but the progression rate is likely to be lower. In this study, among patients with confirmed dengue we will use two definitions of more severe disease: 1) severe dengue as defined in the new WHO 2009 classification scheme and 2) the combined endpoint of hospitalization or IV fluid administration or severe dengue. From a total enrolment of 7–8,000 cases we anticipate that around 3,000 dengue cases will be included in the cohort, potentially with 100–150 patients progressing to severe disease, primarily dengue shock syndrome. Hospitalization and/or IV fluid administration are likely to be considerably more common but will vary according to resources available and local clinical guidelines; however it seems plausible that the *relative* effect of risk factors on outcome will be similar across countries and regions. A dengue cohort of this size should allow for the assessment of up to 15 candidate risk factors even for the less frequent outcome of severe disease.

#### Development of a robust case definition for dengue

We will assess a broad range of clinical and laboratory parameters in order to develop a robust clinical case definition for dengue; covariates assessed will include demographic information, symptoms at presentation, examination findings, and readily available laboratory parameters such as the haematocrit, white blood cell count, lymphocyte count, platelet count etc. as well as simple biochemical measures such as AST, ALT, plasma albumin etc. Development of the diagnostic algorithm will follow standard recommendations for clinical prediction models [29]. Logistic regression will be the primary statistical model but we will also investigate modern flexible classification algorithms such as classification trees and random forests [30]. We anticipate that early changes in serial haematological markers, in particular the platelet count, white blood count and/or lymphocyte count may facilitate diagnosis. Therefore more complex models that include changes in marker values between successive days will also be investigated.

The presumptive case definition for dengue versus OFI may vary according to the background endemicity of other diseases, and we will investigate whether stratification by country or region is necessary. Potential heterogeneity will be assessed by including a fixed continent effect and random country/site effects (mixed effects model), and by testing for covariate-continent/country interactions (fixed effects model) in the logistic model [29].

The main aim for this section of the work is to develop a dengue case definition that can be reliably used at the primary healthcare level, but we may also be able to suggest modifications that could be incorporated into the algorithm if used at higher level healthcare facilities or in middle income countries with more resources available at the primary level. If appropriate the final model will be simplified to a point scoring system or electronic application for direct clinical use [31].

#### Prediction of risk for progression to severe dengue

Among patients with confirmed dengue we will identify simple clinical and laboratory parameters associated with either of the two definitions of more severe disease described above. Heterogeneity of outcomes will be assessed prior to pooling datasets from different countries. Classical prognostic models depend on baseline covariates only. However, we anticipate that early changes in serial haematological markers, in particular the platelet count, white blood count and/or lymphocyte count, or potentially in clinical parameters may carry important additional prognostic information. Thus there is a need to incorporate longitudinal data into the prognostic models and to update the models based on incoming data – e.g. by developing partly conditional models or joint models of longitudinal markers and outcome [32, 33].

#### Identification of early virological correlates of severe dengue

We will test the hypothesis that viral markers associated with development of severe dengue can be identified within the first 3 days of fever. We will define the sensitivity, specificity, positive and negative predictive values of NS1 detection within 3 days of fever onset for the same severity measures as indicated above and will also explore the use of statistical models for severity prediction.

#### Identification of early serological correlates of severe dengue

We will assess a variety of antibody responses (neutralizing and enhancing titers against the 4 dengue serotypes plus antibody titers against E, DIII, prM and NS1 of the 4 serotypes) in relation to clinical outcome, plasma viremia and NS1 antigenemia, and will develop a model of the serological responses seen in order to identify correlates of severe disease.

### Ethical considerations

This research project follows international standards for the ethical conduct of research involving human subjects. Ethical clearance has been obtained from the responsible institutional and national boards for each participating country (Table 1). Prior to commencing the study 3-day workshops were arranged in each country to provide a) general training in research ethics and ICH-GCP and b) specific training on the study protocol for all staff, as well as to allow development of locally appropriate SOPs for enrolment, daily follow-up, sample management etc. together with designated staff members. All staff involved in the study completed the relevant training before the study commenced at their site, and additional courses are being provided at intervals to ensure GCP training is maintained up to date.

#### Informed consent

Written informed consent is obtained from all patients or from the parent/guardian of children, as described above in the section on patient enrolment.

#### Confidentiality

Participants are assured that all information generated in this study will remain confidential. All data (including clinical, laboratory and genetic data) are stored in password-protected databases. Participants’ names are recorded at the time of enrolment to allow for their identification at follow-up visits, but identifiable information is linked to stored data or samples only by a protected Master List. This list is not shared outside the study staff at a unique hospital, and no identifying information is transferred between sites. All CRFs and samples are labelled with a study identification number only and stored in suitable secure locations. Only persons who have signed the locally appropriate data protection commitment form have access to the password-protected computer where entered data is stored. After conclusion of the project data will be removed from the computers and stored in a safe place.

#### Diagnostic tests

This is a prospective observational study in which dengue diagnostic tests are performed on plasma samples from suspected dengue patients. The diagnostic tests are not performed in real time but batch tested at intervals; thus neither the patient nor the study/attending doctors are given the results of the tests as we intend to evaluate their accuracy in identifying patients who progress to severe dengue without the bias introduced by physicians interpreting and making clinical management decisions based on these test results. The routine diagnostic tests performed at each site are available to the treating clinicians as per the local standard of care. The research diagnostic tests are not routine in these settings and therefore we are not altering the current standard of care in the outpatient setting.

#### Genetic testing, export and storage of samples

We ask participants (or their parents/guardians) to consent to having blood samples stored, to export samples, and potentially to have genetic studies performed on the study participants’ DNA. All stored and exported samples are pseudonymized and linked to protected identifying information at the study site only. No identifying data is exported or shared. Any genetic studies performed are exploratory and it is made clear to participants that we do not know whether the results will be helpful in treating patients with this disease.

#### Participant remuneration

We ensure that study participation imposes no cost implications for the patient or family. All research tests and study medical consultations are paid for by the study, and travel costs at appropriate local rates are reimbursed for attendance at the daily follow-up visits. No other financial incentives are offered.

#### Exclusion of children under 5 years

The sample size calculation is based on approximately 40 % of patients enrolled to the study testing positive for dengue. In children less than 5 years, the number of confirmed dengue infections among patients presenting with undifferentiated fever is much lower than in the rest of the population due to the high incidence of other infections. By excluding children less than 5 years old, the desired sample size can be met with a lower overall enrolment and fewer small children being exposed to the risks and inconvenience of participation/blood sampling.

#### Local health resources

Dedicated study staff are funded by the program to execute study related procedures in order to minimise the burden of the research on local health staff and facilities. In addition study staff help to reduce the regular workload of clinic staff by contributing to standard care activities.

## Discussion

Differentiating dengue from other common febrile illnesses before complications develop is difficult. Simple and inexpensive strategies are urgently needed to support early and accurate diagnosis, as well as to identify patients at high risk of developing complications, in order both to improve case management and to facilitate appropriate use of limited resources. Evaluation of early clinical features alongside readily available laboratory tests in a large cohort of patients encompassing the breadth of dengue disease encountered in endemic settings is necessary to develop a robust case definition for dengue, and could also prove to be very useful for the development of prognostic algorithms. Similarly, characterisation of the profiles of important viral and serological biomarkers in such a cohort is likely to provide valuable information that could contribute additional input into diagnostic and prognostic algorithms. Improved strategies for early diagnosis and risk prediction would also enhance the conduct of clinical trials of early therapeutic interventions for dengue e.g. use of anti-viral or immunomodulatory drugs. Finally preliminary evidence indicates associations between certain genetic markers and severe dengue; this cohort should provide a valuable resource to investigate these genetic associations in more detail.

### Timeline

The study commenced at one site in Vietnam in October 2011 and was gradually extended to the other participating countries such that all sites were actively recruiting by September 2014. As of December 2015 a total of 7096 participants had been enrolled, with dengue confirmed in 2510/5996 (42 %) of the cases for which dengue diagnostics are available at this time. Staggered close out is planned over the coming 6 months, with all sites expected to complete enrolment by June 2016. A formal data analysis plan has been written, and full analysis/interpretation, dissemination of the findings, and report writing are scheduled to take place by the end of 2016.

## Abbreviations

ALT: Alanine aminotransferase
AST: Aspartate aminotransferase
CRF: Case report form
DF: Dengue fever
DENV: Dengue Virus
DHF: Dengue haemorrhagic fever
DIII: Envelope protein domain 3
DSS: Dengue shock syndrome
E: Envelope protein
GCP: Good clinical practice
HCMC: Ho Chi Minh City
NS1: Non-structural protein 1
OFI: Other febrile illness
OPD: Outpatient department
prM: Pre-membrane protein
SOP: Standard operating procedures
